# Systemic inflammatory indices and 28-day mortality in severe pneumonia: a retrospective single-center exploratory study

**DOI:** 10.3389/fmed.2026.1787128

**Published:** 2026-04-21

**Authors:** Xingxing Chen, Weiqiang Huang, Wenjing Dai, Wei Zhang, Xiaofeng Zhong, Ming Hu

**Affiliations:** Intensive Care Unit, Wuhan Pulmonary Hospital, Wuhan, Hubei, China

**Keywords:** immune-inflammatory biomarkers, inflammation-based index, intensive care unit, prognostic model, risk stratification, severe pneumonia

## Abstract

**Background:**

Severe pneumonia remains a major cause of intensive care unit admission and death, and practical tools for early risk stratification remain limited. We evaluated several readily available systemic inflammatory indices and their association with 28-day all-cause mortality in patients with severe pneumonia.

**Methods:**

This retrospective single-center study included 100 ICU patients with severe pneumonia treated between January 2022 and December 2023. The primary endpoint was 28-day all-cause mortality. Patients were classified into a death group (*n* = 37) and a non-death group (*n* = 63). Systemic immune-inflammation index (SII), systemic inflammatory response index (SIRI), neutrophil-to-lymphocyte ratio (NLR), platelet-to-lymphocyte ratio (PLR), and inflammatory burden index (IBI) were calculated from routine laboratory data. Logistic regression models with progressive adjustment were used to evaluate the independent associations of these indices with 28-day mortality.

**Results:**

Several inflammatory indices were higher in patients who died within 28 days. In unadjusted and minimally adjusted analyses, SIRI, NLR, and IBI were significantly associated with 28-day mortality. After further adjustment for age, sex, body mass index, smoking, drinking, admission APACHE II score, oxygenation index, mechanical ventilation duration, and major baseline comorbidities, only SIRI and NLR remained independently associated with 28-day mortality. In the fully adjusted model, each 1-standard deviation increase in SIRI and NLR was associated with higher odds of 28-day death (SIRI: OR 2.16, 95% CI 1.08–4.33; NLR: OR 2.12, 95% CI 1.14–3.95). By contrast, the associations of SII and IBI were attenuated after additional adjustment, and PLR was not independently associated with 28-day mortality.

**Conclusion:**

Among the evaluated inflammatory indices, SIRI and NLR showed the most stable independent associations with 28-day all-cause mortality in severe pneumonia. These findings are exploratory and require external validation, but they suggest that selected inflammation-based indices may provide additional prognostic information beyond conventional severity markers.

## 1 Introduction

Severe pneumonia remains a major cause of intensive care unit admission and short-term death, particularly in older adults and in patients with substantial physiological derangement or immunologic vulnerability (1–6). Although established severity tools such as APACHE II and oxygenation-related indices are clinically useful, they do not fully capture the host inflammatory response that accompanies severe pulmonary infection (7–12). Because routine blood tests are rapidly available at admission, inflammation-based composite indices have attracted increasing interest as practical candidates for early risk stratification in pneumonia and other acute illnesses (13–16).

Several studies have already explored these indices in different pneumonia settings. Prior work has examined the clinical value of NLR and related indices in pneumonia (17, 18), whereas SIRI has also been investigated in necrotizing pneumonia and postoperative pneumonia prediction (19, 20). Additional studies have evaluated NLR, SII, and PLR in *Mycoplasma pneumoniae*, COVID-19, and community-acquired pneumonia (21–24). Evidence for IBI has been described more clearly in other inflammatory or high-risk clinical settings (25–27). In community-acquired pneumonia, Yeşildağ et al. specifically evaluated the relationship between biomarkers and disease severity (28). More recently, Yeşildağ and Kocabas compared new hematologic indices between Influenza A (H1N1) and COVID-19 pneumonias, further showing that inflammatory-marker profiles may differ across pneumonia phenotypes (29). Additional CAP studies have also reported that serial NLR measurement may help predict short-term prognosis and early treatment response in hospitalized patients (30), that broader biomarker panels including NLR, SII, and SIRI may differ between survivors and non-survivors (31), and that SII may show a stronger short-term mortality signal than SIRI when evaluated alongside established severity scores (32).

At the same time, the existing literature also suggests that these markers should not be viewed as interchangeable. Some studies have reported favorable prognostic associations for NLR or related indices in selected pneumonia populations (17–24), whereas evidence for IBI remains stronger in other inflammatory or high-risk clinical settings (25–27). More recent CAP analyses have extended this literature (28–32), but the strength and consistency of these associations still vary across age groups, pneumonia subtypes, outcome definitions, and analytical strategies. For example, serial NLR appears informative in hospitalized CAP (30), while comparative analyses suggest that the incremental contribution of SII or SIRI beyond conventional severity scores may be modest and context-dependent (31, 32). Therefore, positive findings from one pneumonia phenotype cannot be assumed to apply directly to ICU patients with severe pneumonia, and the independent contribution of each composite index still requires clarification in this setting.

In this study, we investigated the prognostic value of a panel of composite inflammatory indices, including SII, SIRI, NLR, PLR, and IBI, in ICU patients with severe pneumonia. By evaluating these indices within the same cohort and then progressively adjusting for demographic characteristics, illness severity, and major baseline comorbidities, we aimed to determine which markers retained the most stable association with 28-day all-cause mortality. This design was intended to provide a more clinically grounded and internally comparative assessment of inflammation-based prognostic indices in severe pneumonia.

## 2 Materials and methods

### 2.1 Study population and design

This study was conducted to evaluate the associations of systemic inflammatory indices with 28-day all-cause mortality in patients with severe pneumonia. A total of 100 patients were included in the analysis, all of whom were admitted to the ICU of a tertiary hospital between January 2022 and December 2023. The inclusion criteria were based on the American Thoracic Society/Infectious Diseases Society of America (ATS/IDSA) guidelines, which define severe pneumonia by criteria such as the need for mechanical ventilation, septic shock, or multi-organ dysfunction. Eligible patients were older than 18 years with a confirmed diagnosis of severe pneumonia based on clinical and radiographic findings. Patients with incomplete medical records, those who were transferred from other hospitals, and those with a known history of immunodeficiency disorders were excluded from the study. The study protocol was reviewed and approved by the ethical committee of Wuhan Pulmonary Hospital, Wuhan, Hubei, China; approval was obtained prior to data extraction and analysis. The study was conducted in accordance with the Declaration of Helsinki. Given the retrospective design and the use of routinely collected, de-identified clinical data, the ethics committee/IRB waived the requirement for informed consent.

### 2.2 Data acquisition process

Data were retrospectively collected from electronic medical records and included demographic characteristics [age, sex, body mass index (BMI)], lifestyle factors (current smoking and current drinking status), clinical severity indicators (admission APACHE II score, oxygenation index (OI, defined as PaO_2_/FiO_2_), and mechanical ventilation duration), and laboratory parameters used to derive systemic inflammatory indices. The primary outcome was 28-day all-cause mortality, defined according to the 28-day follow-up record. Length of stay was recorded descriptively but was not included as a primary study endpoint in the present analysis.

### 2.3 Grouping and conversion of indicators

All patients were classified into two groups according to 28-day follow-up status: the death group (*n* = 37) and the non-death group (*n* = 63). The non-death group included patients who were alive at 28 days. Systemic inflammatory indices were calculated from routinely collected laboratory data as follows: SII = platelet count × neutrophil count/lymphocyte count; SIRI = neutrophil count × monocyte count/lymphocyte count; IBI = C-reactive protein × neutrophil count/lymphocyte count; NLR = neutrophil count/lymphocyte count; and PLR = platelet count/lymphocyte count.

### 2.4 Statistical analysis

All descriptive analyses were performed using SPSS version 26.0 and R version 4.2.3. Continuous variables were presented as mean ± standard deviation or median (interquartile range), as appropriate, and categorical variables were presented as frequencies and percentages. The primary analysis was conducted in 100 patients with available 28-day follow-up data. After excluding patients with incomplete medical records, there were no missing values for variables included in the primary analyses in the final analysis dataset; therefore, complete-case analysis was performed without imputation.

The associations between inflammatory indices and 28-day all-cause mortality were evaluated using logistic regression. Model 1 was unadjusted. Model 2 was adjusted for age, sex, BMI, current smoking, and current drinking. Model 3 was further adjusted for admission APACHE II score, oxygenation index, and mechanical ventilation duration. Model 4 was additionally adjusted for baseline hypertension, diabetes mellitus, chronic obstructive pulmonary disease, and coronary heart disease. All inflammatory indices were standardized as z scores before regression.

To further explore model performance, core exploratory models were constructed by combining admission APACHE II score, oxygenation index, and mechanical ventilation duration with one inflammatory index at a time. Model fit was compared using the Akaike information criterion, and discriminative performance was summarized using apparent area under the receiver operating characteristic curve.

A two-sided *p* < 0.05 was considered statistically significant.

## 3 Results

### 3.1 Demographic and clinical characteristics

A total of 100 patients were included in this analysis, with a majority being male (58%, *n* = 58), as shown in Table 1. The median age of the participants was 68.0 years (IQR: 59.0–76.25 years), indicating a predominantly elderly patient population. The median BMI was 18.66 kg/m^2^ (IQR: 16.27–22.05 kg/m^2^). Notably, there was a statistically significant difference between the non-death and death groups in mechanical ventilation duration (*p* = 0.021) and APACHE II score (*p* < 0.001). Regarding baseline characteristics, current smoking was reported in 38% of the patients, while current drinking was noted in 17%. Hypertension affected 29% of the cohort, diabetes mellitus affected 29%, and both coronary heart disease and chronic obstructive pulmonary disease (COPD) were present in 22% of the cohort.

**Table 1:** Demographic and clinical characteristics of the participants (*n* = 100).

Variables	Total	Non-death group	Death group	*p*-value
*N*	100	63	37	
Male, n (%)	58 (58.0)	39 (61.9)	19 (51.4)	0.302
Age (years)	68.00 (59.00, 76.25)	66.00 (59.00, 76.00)	70.00 (58.00, 79.00)	0.658
BMI (kg/m^2^)	18.66 (16.27, 22.05)	17.96 (15.78, 21.20)	19.05 (16.65, 23.15)	0.283
LOS (days)	12.00 (4.94, 22.25)	10.00 (5.72, 23.00)	13.00 (4.75, 20.72)	0.946
Mechanical ventilation time (days)	4.00 (0.00, 16.00)	1.00 (0.00, 15.00)	9.00 (1.00, 16.00)	0.021
APACHE II	21.00 (16.75, 26.00)	18.00 (14.50, 23.50)	24.00 (21.00, 29.00)	<0.001
Smoking, n (%)	38 (38.00)	26 (41.27)	12 (32.43)	0.379
Drinking, n (%)	17 (17.00)	11 (17.46)	6 (16.22)	0.873
Hypertension, n (%)	29 (29.00)	17 (26.98)	12 (32.43)	0.562
Coronary heart disease, n (%)	22 (22.00)	15 (23.81)	7 (18.92)	0.569
Diabetes, n (%)	29 (29.00)	18 (28.57)	11 (29.73)	0.902
COPD, n (%)	22 (22.00)	13 (20.63)	9 (24.32)	0.667

Continuous variables are presented as median (IQR). Patients were categorized according to 28-day follow-up status. PCT, procalcitonin; CRP, C-reactive protein; WBC, white blood cell count; NEU, neutrophil count; LYM, lymphocyte count; PLT, platelet count; Fib, fibrinogen; ALT, alanine transaminase; AST, aspartate transaminase; Lac, lactate. The significance of bold indicates that the p value is less than 0.05.

As shown in Table 2, the overall median PCT concentration was 0.35 ng/mL (IQR: 0.12, 1.29), with no significant difference between the non-death and death groups (*p* = 0.867). WBC count, platelet count, fibrinogen, alanine transaminase, aspartate transaminase, and D-dimer did not differ significantly between the two groups. Neutrophil count was significantly higher in the death group (median: 9.67 × 10^9/L; IQR: 6.74, 13.17) than in the non-death group (median: 6.75 × 10^9/L; IQR: 4.75, 10.55) (*p* = 0.025). Conversely, lymphocyte count was significantly lower in the death group (median: 0.470 × 10^9/L; IQR: 0.260, 0.770) than in the non-death group (median: 0.760 × 10^9/L; IQR: 0.460, 1.270) (*p* = 0.008). The PaO2/FiO2 ratio was also significantly lower in the death group (median: 71.00; IQR: 53.00, 121.25) than in the non-death group (median: 158.00; IQR: 83.88, 264.00) (*p* < 0.001). Lactate tended to be higher in the death group (median: 1.80 mmol/L; IQR: 1.20, 2.85) than in the non-death group (median: 1.40 mmol/L; IQR: 1.00, 1.95), although this difference did not reach statistical significance (*p* = 0.065).

**Table 2:** Clinical characteristics of the participants (*n* = 100).

Variables	Total	Non-death group	Death group	*P*-value
*N*	100	63	37	
PCT (ng/mL)	0.35 (0.12, 1.29)	0.35 (0.12, 1.54)	0.31 (0.17, 0.91)	0.867
CRP (mg/L)	75.33 (24.51, 118.54)	52.10 (16.95, 115.56)	91.42 (38.00, 124.20)	0.062
WBC (×10^9^/L)	9.01 (6.66, 13.53)	8.54 (6.55, 12.75)	11.33 (7.14, 14.10)	0.337
NEU (×10^9^/L)	7.26 (5.14, 11.55)	6.75 (4.75, 10.55)	9.67 (6.74, 13.17)	0.025
LYM (×10^9^/L)	0.680 (0.370, 1.165)	0.760 (0.460, 1.270)	0.470 (0.260, 0.770)	0.008
PLT (×10^9^/L)	205.00 (123.00, 262.25)	214.00 (127.00, 271.50)	198.00 (117.00, 239.00)	0.323
D-dimer (μg/L)	2.72 (0.95, 6.09)	2.60 (0.87, 5.75)	2.78 (1.15, 7.01)	0.270
Fib (g/L)	3.15 (2.27, 4.39)	3.15 (2.17, 4.67)	3.03 (2.48, 3.98)	0.817
ALT (U/L)	17.50 (10.00, 40.25)	15.00 (9.00, 33.50)	23.00 (11.00, 50.00)	0.108
AST (U/L)	28.50 (18.00, 64.00)	25.00 (16.50, 54.50)	35.00 (19.00, 77.00)	0.119
PaO_2_/FiO_2_	119.29 (68.25, 226.00)	158.00 (83.88, 264.00)	71.00 (53.00, 121.25)	<0.001
Lac (mmol/L)	1.50 (1.08, 2.50)	1.40 (1.00, 1.95)	1.80 (1.20, 2.85)	0.065

### 3.2 Comparison of systemic inflammatory indices between the non-death and death groups of patients with severe pneumonia

**Figure 1:**
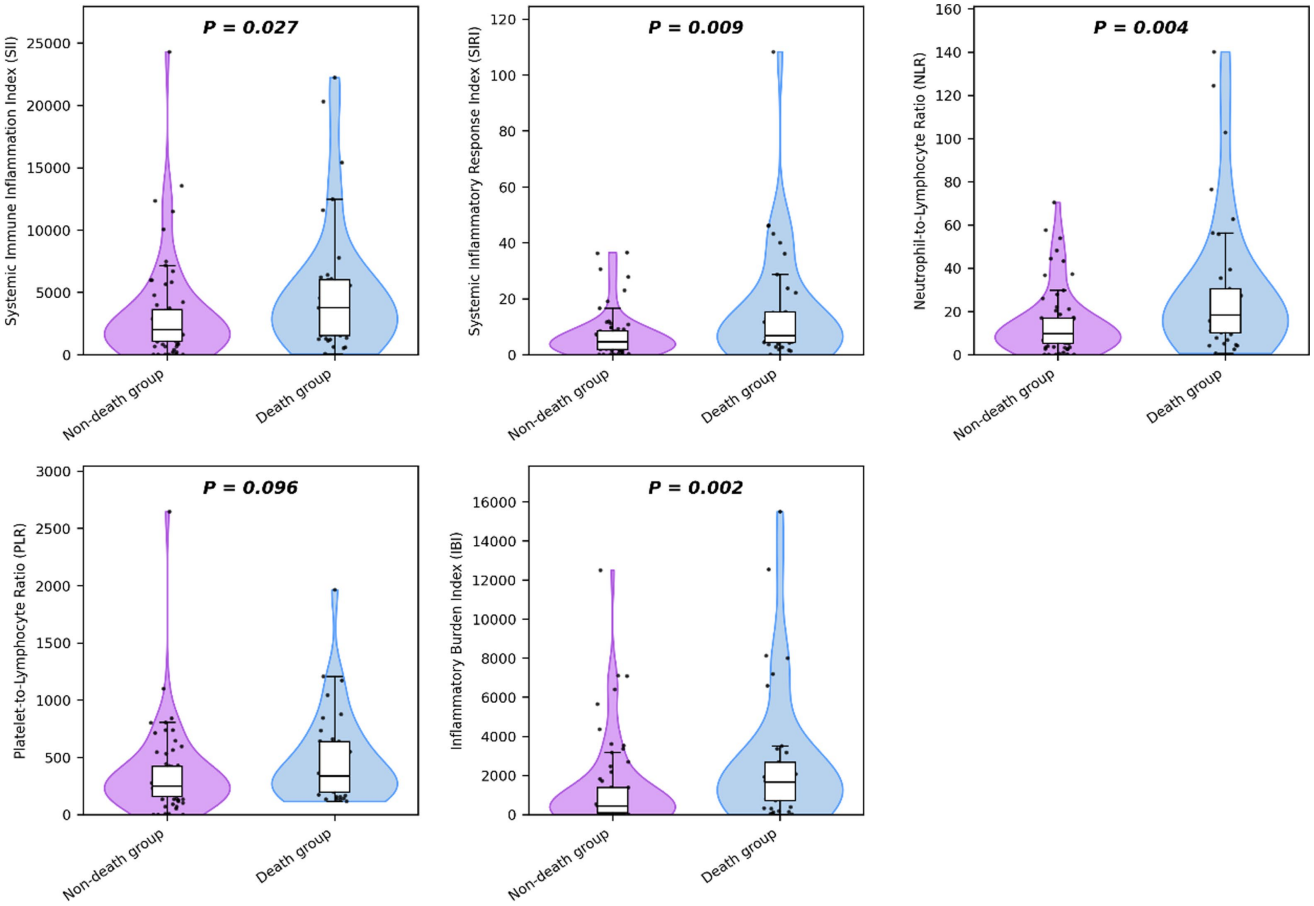
Comparison of systemic inflammatory indices between the non-death and death groups.

Figure 1 displays the comparison of systemic inflammatory indices between the non-death and death groups of patients with severe pneumonia. SII was significantly higher in the death group (median: 3755.20; IQR: 1533.75, 6028.53) than in the non-death group (median: 2004.12; IQR: 1079.12, 3635.93) (*p* = 0.027) (Table 3). Similarly, SIRI was significantly higher in the death group (median: 6.79; IQR: 4.40, 15.35) than in the non-death group (median: 4.62; IQR: 1.97, 8.47) (*p* = 0.009). NLR was also significantly higher in the death group (median: 18.37; IQR: 10.18, 30.64) than in the non-death group (median: 9.61; IQR: 5.28, 17.07) (*p* = 0.004). PLR did not show a significant difference between the two groups (*p* = 0.096). IBI was significantly higher in the death group (median: 1668.21; IQR: 724.20, 2685.27) than in the non-death group (median: 433.64; IQR: 94.75, 1381.55) (*p* = 0.002).

**Table 3:** Comparison of systemic inflammatory indices between the non-death and death groups of patients with severe pneumonia.

Variables	Total	Non-death group	Death group	*P*-value
SII	2386.37 (1214.36, 4621.32)	2004.12 (1079.12, 3635.93)	3755.20 (1533.75, 6028.53)	**0.027**
SIRI	5.29 (2.62, 10.32)	4.62 (1.97, 8.47)	6.79 (4.40, 15.35)	**0.009**
NLR	12.59 (6.44, 26.03)	9.61 (5.28, 17.07)	18.37 (10.18, 30.64)	**0.004**
PLR	294.58 (174.85, 534.79)	249.48 (158.66, 421.86)	336.62 (196.83, 638.71)	0.096
IBI	848.44 (147.47, 2090.87)	433.64 (94.75, 1381.55)	1668.21 (724.20, 2685.27)	**0.002**

Values are presented as median (IQR). Patients were categorized into the non-death and death groups according to 28-day follow-up status. SII, systemic immune-inflammation index; SIRI, systemic inflammatory response index; NLR, neutrophil-to-lymphocyte ratio; PLR, platelet-to-lymphocyte ratio; IBI, inflammatory burden index. The bold values indicate that the *p* value is less than 0.05.

**Figure 2:**
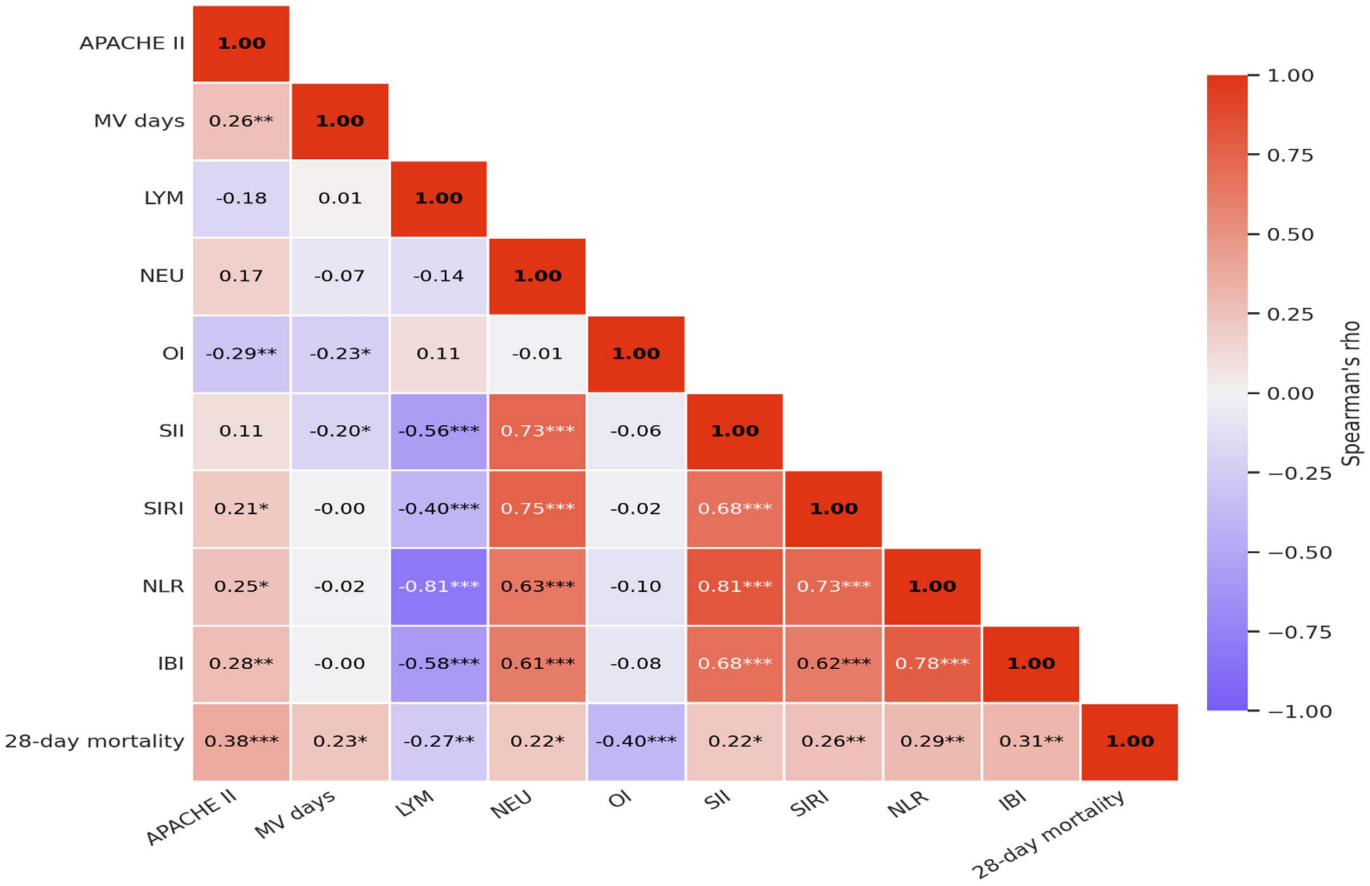
Spearman correlation matrix of 28-day all-cause mortality and key clinical and inflammatory indicators (*n* = 100). Note: Cell color represents Spearman’s rho. Mortality was coded as 1 for death and 0 for non-death. Asterisks indicate statistical significance (*p < 0.05, **p < 0.01, ***p < 0.001).

Figure 2 presents the Spearman correlation matrix for 28-day all-cause mortality and key clinical and inflammatory indicators. Higher APACHE II score, mechanical ventilation duration, neutrophil count, SII, SIRI, NLR, and IBI were positively correlated with 28-day mortality, whereas lymphocyte count and oxygenation index were negatively correlated.

### 3.3 Associations of inflammatory indices with 28-day all-cause mortality

**Figure 3:**
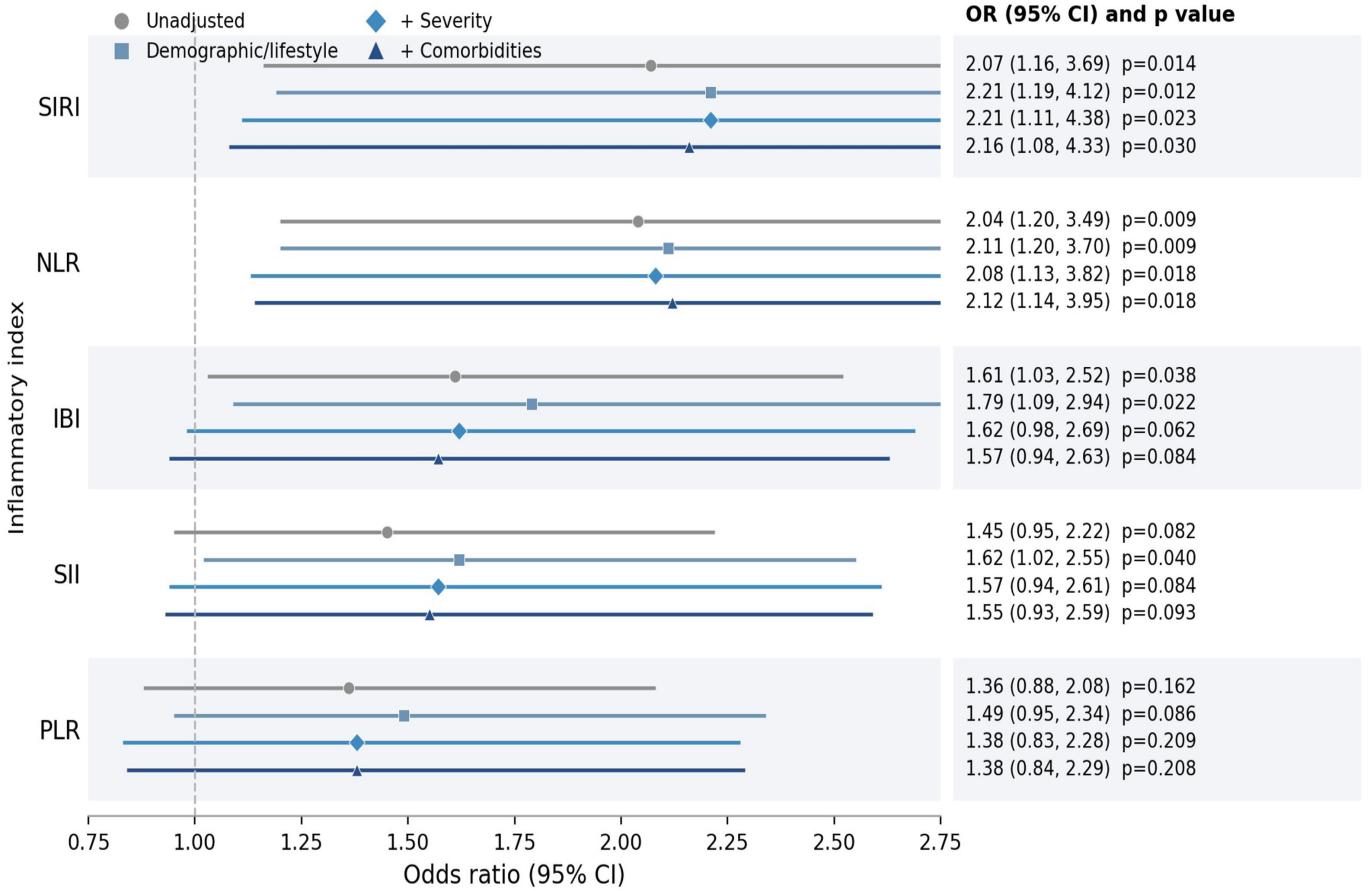
Forest plot of the associations between inflammatory indices and 28-day all-cause mortality across progressively adjusted logistic regression models. Odds ratios and 95% confidence intervals are shown for Model 1 (unadjusted), Model 2 (adjusted for age, sex, body mass index, current smoking, and current drinking), Model 3 (further adjusted for admission APACHE II score, oxygenation index, and mechanical ventilation duration), and Model 4 (further adjusted for baseline hypertension, diabetes mellitus, chronic obstructive pulmonary disease, and coronary heart disease).

To further evaluate the prognostic value of inflammatory indices, we performed logistic regression analyses using 28-day all-cause mortality as the dependent variable (Table 4). The corresponding forest plot summarizing these progressively adjusted associations is shown in Figure 3. In the unadjusted analyses, higher SIRI, NLR, and IBI were significantly associated with increased odds of 28-day death. After adjustment for age, sex, BMI, smoking, and drinking status (Model 2), these associations remained significant, and SII also reached statistical significance. However, after further adjustment for admission APACHE II score, oxygenation index, and mechanical ventilation duration (Model 3), only SIRI and NLR remained independently associated with 28-day mortality. In the fully adjusted model additionally including baseline hypertension, diabetes mellitus, COPD, and coronary heart disease (Model 4), the associations for SIRI and NLR remained statistically significant, whereas those for SII, PLR, and IBI were attenuated and no longer reached conventional statistical significance.

**Table 4:** Associations of inflammatory indices with 28-day all-cause mortality in patients with severe pneumonia (*n* = 100).

Predictor	Model 1 OR (95% CI); *P*	Model 2 OR (95% CI); *P*	Model 3 OR (95% CI); *P*	Model 4 OR (95% CI); *P*
SII	1.45 (0.95–2.22) *p* = 0.082	1.62 (1.02–2.55) *p* = 0.040	1.57 (0.94–2.61) *P* = 0.084	1.55 (0.93–2.59) *p* = 0.093
SIRI	2.07 (1.16–3.69) *p* = 0.014	2.21 (1.19–4.12) *p* = 0.012	2.21 (1.11–4.38) *P* = 0.023	2.16 (1.08–4.33) *P* = 0.030
NLR	2.04 (1.20–3.49) *p* = 0.009	2.11 (1.20–3.70) *p* = 0.009	2.08 (1.13–3.82) *p* = 0.018	2.12 (1.14–3.95) *P* = 0.018
PLR	1.36 (0.88–2.08) *p* = 0.162	1.49 (0.95–2.34) *p* = 0.086	1.38 (0.83–2.28) *p* = 0.209	1.38 (0.84–2.29) *p* = 0.208
IBI	1.61 (1.03–2.52) *p* = 0.038	1.79 (1.09–2.94) *p* = 0.022	1.62 (0.98–2.69) *p* = 0.062	1.57 (0.94–2.63) *p* = 0.084

Model 1 was unadjusted. Model 2 was adjusted for age, sex, body mass index (BMI), current smoking, and current drinking. Model 3 was further adjusted for admission APACHE II score, oxygenation index (OI, defined as PaO_2_/FiO_2_), and mechanical ventilation duration. Model 4 was further adjusted for baseline hypertension, diabetes mellitus, chronic obstructive pulmonary disease (COPD), and coronary heart disease (CHD). All inflammatory indices were standardized as z scores before regression. Odds ratios therefore represent the association per 1-standard deviation increase in each inflammatory index. SII, systemic immune-inflammation index; SIRI, systemic inflammatory response index; NLR, neutrophil-to-lymphocyte ratio; PLR, platelet-to-lymphocyte ratio; IBI, inflammatory burden index; APACHE II, Acute Physiology and Chronic Health Evaluation II; OI, oxygenation index; BMI, body mass index; COPD, chronic obstructive pulmonary disease; CHD, coronary heart disease.

Specifically, each 1-standard deviation increase in SIRI was associated with a 2.16-fold higher odds of 28-day death in Model 4 (OR 2.16, 95% CI 1.08–4.33, *p* = 0.030). Similarly, each 1-standard deviation increase in NLR was associated with a 2.12-fold higher odds of 28-day death in Model 4 (OR 2.12, 95% CI 1.14–3.95, *p* = 0.018). In contrast, the association of IBI was significant in the less adjusted models but weakened after additional adjustment for severity-related and comorbidity variables (Model 4: OR 1.57, 95% CI 0.94–2.63, *p* = 0.084). A similar attenuation pattern was observed for SII, while PLR was not significantly associated with 28-day mortality in any model.

### 3.4 Robustness of inflammatory indices after progressive adjustment

**Figure 4:**
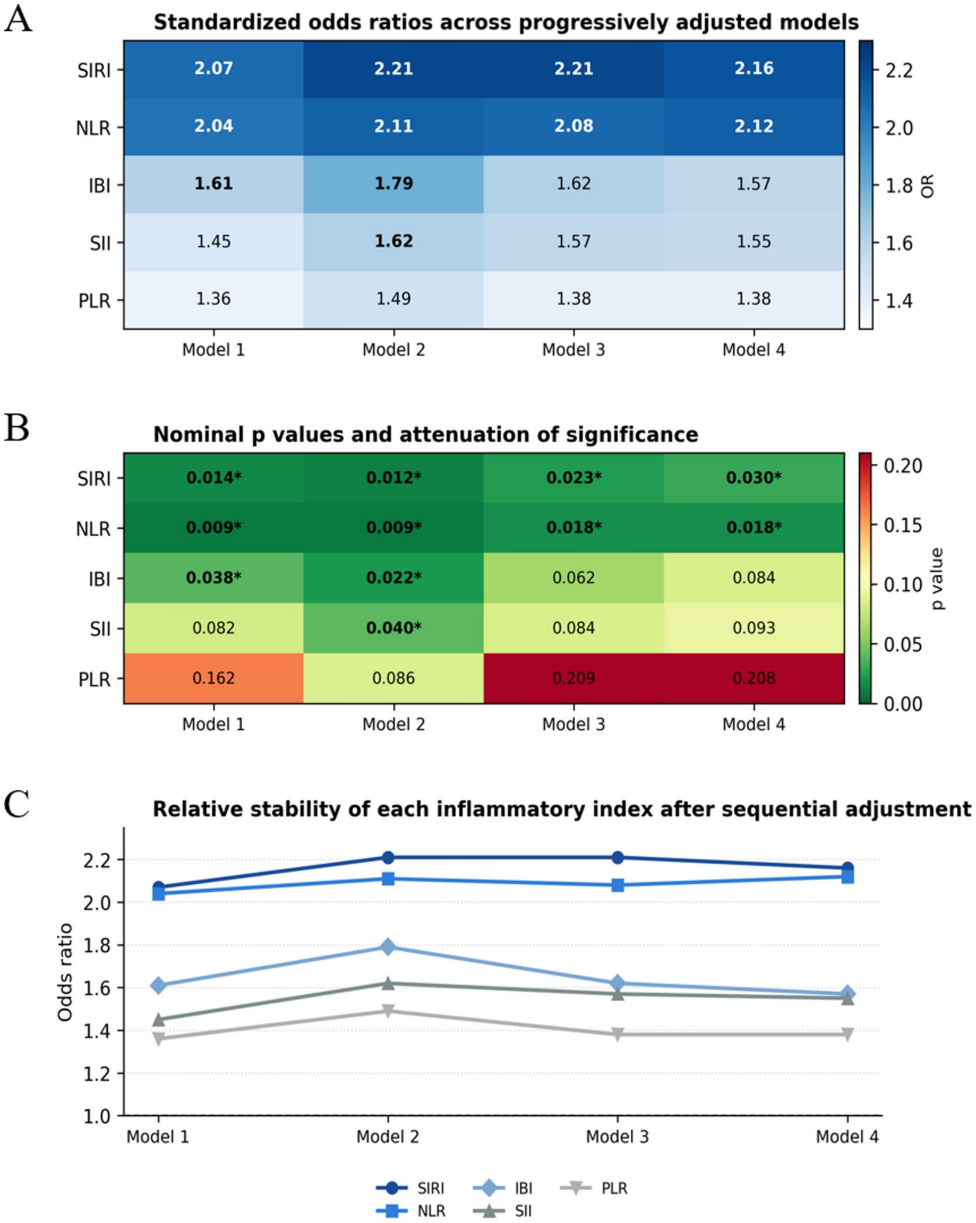
Visual summary of the progressive attenuation of inflammatory indices across sequentially adjusted models. **(A)** Heatmap of standardized odds ratios across Models 1–4. **(B)** Heatmap of nominal *p* values; cells marked with an asterisk indicate *p* < 0.05. **(C)** Line plot showing the relative stability of each inflammatory index after sequential adjustment for demographic/lifestyle factors, illness severity, and baseline comorbidities.

A progressive attenuation pattern was observed for several inflammatory indices after sequential adjustment for demographic characteristics, illness severity, and baseline comorbidities. Among the five candidate indices, SIRI and NLR showed the most stable associations across all nested models. By contrast, SII and IBI appeared to be more sensitive to confounding by disease severity and comorbidity burden, as their initial associations were weakened after the inclusion of APACHE II score, oxygenation index, mechanical ventilation duration, and major baseline comorbidities. PLR showed no significant association throughout the analysis. A visual summary of this attenuation pattern is shown in Figure 4.

These findings suggest that not all composite inflammatory indices provide the same degree of independent prognostic information in severe pneumonia. Rather, the results support a more selective interpretation in which SIRI and NLR may retain incremental prognostic relevance beyond conventional markers of illness severity, whereas the observed associations of SII and IBI appear to be at least partly explained by differences in baseline severity and comorbidity structure.

### 3.5 Core exploratory models for 28-day all-cause mortality

**Figure 5:**
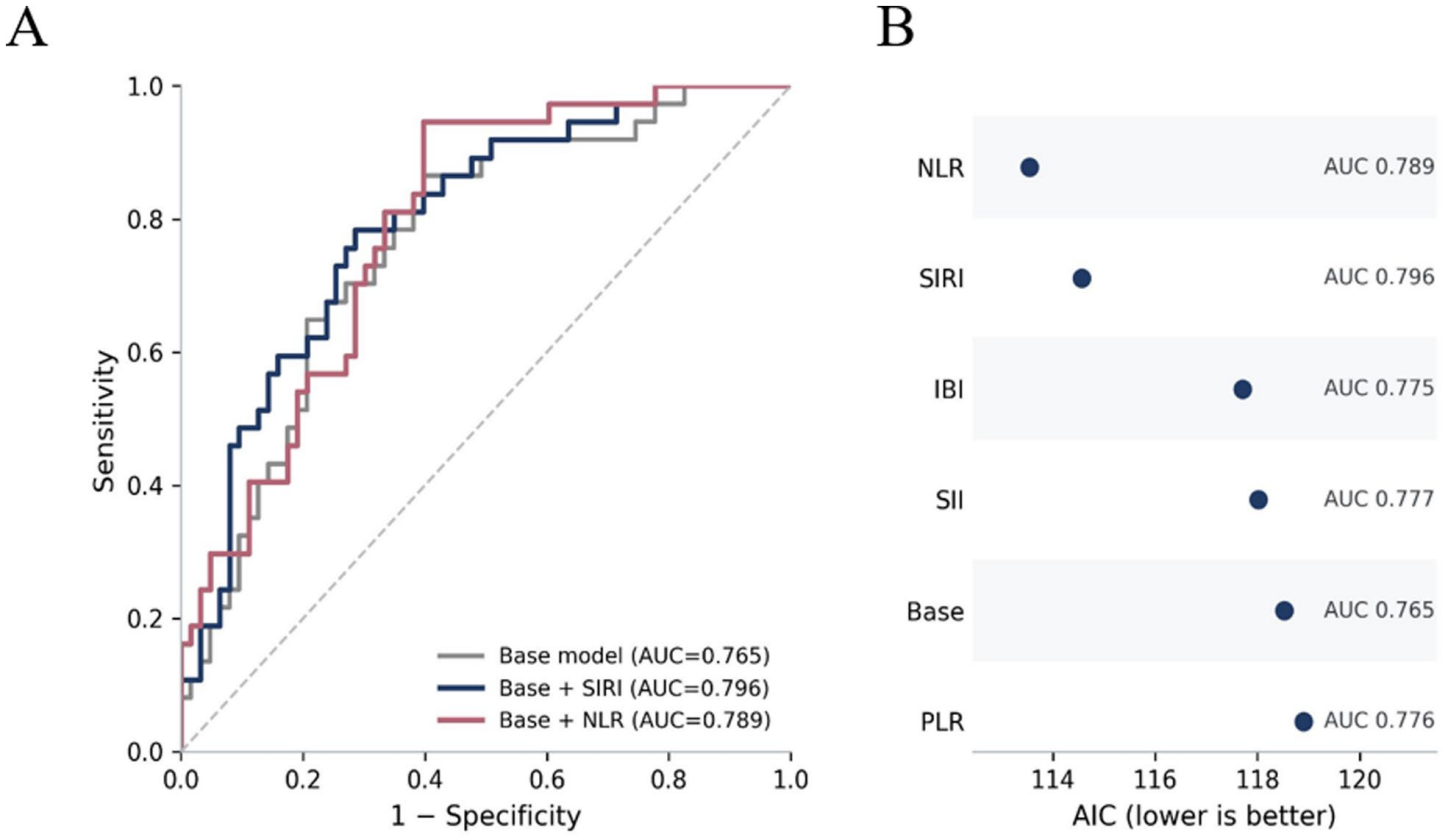
Core exploratory models for 28-day all-cause mortality. **(A)** ROC curves for the base, base + SIRI, and base + NLR models. **(B)** Comparison of apparent AUC and AIC across the core exploratory models.

Given the limited event number in this single-center cohort, we further constructed a set of core exploratory models that included admission APACHE II score, oxygenation index, mechanical ventilation duration, and one inflammatory index at a time. The ROC and model-comparison summaries of these exploratory models are shown in Figure 5. In these severity-adjusted exploratory models, the best-performing biomarker additions were NLR and SIRI. The model including NLR yielded the lowest AIC (113.54) and an apparent AUC of 0.789, with NLR remaining independently associated with 28-day mortality (OR 1.96, 95% CI 1.10–3.50, *p* = 0.023). The corresponding model including SIRI also performed well (AIC 114.56; apparent AUC 0.796), and SIRI remained significant (OR 1.96, 95% CI 1.05–3.65, *p* = 0.035).

By comparison, the addition of IBI, SII, or PLR did not provide similarly robust evidence of independent association in these severity-adjusted exploratory models. Their *p-*values exceeded 0.05, and model fit was less favorable. Taken together, these analyses indicate that SIRI and NLR may be the most promising inflammatory indices for further validation in severe pneumonia, although the present findings should be interpreted as exploratory and hypothesis-generating rather than definitive.

## 4 Discussion

In this revised analysis using 28-day all-cause mortality as the primary endpoint, the prognostic performance of systemic inflammatory indices differed after progressive adjustment for demographic factors, illness severity, and baseline comorbidities. SIRI and NLR showed the most stable associations with 28-day death, whereas the associations of SII and IBI were attenuated after additional adjustment, and PLR was not independently associated with mortality.

### 4.1 Independent association of inflammatory indices with 28-day mortality

Among the five evaluated indices, SIRI and NLR retained statistically significant associations with 28-day all-cause mortality after adjustment for age, sex, BMI, smoking, drinking, admission APACHE II score, oxygenation index, mechanical ventilation duration, and major baseline comorbidities. By contrast, the associations of SII and IBI were weakened after the inclusion of severity-related and comorbidity variables, suggesting that part of their apparent signal may reflect the underlying burden of acute physiological derangement rather than a fully independent prognostic effect.

This pattern is biologically plausible. Both SIRI and NLR capture a combination of neutrophil-predominant innate immune activation and relative lymphocyte suppression, which are closely linked to dysregulated host response in severe infection and respiratory failure. Our findings are also broadly consistent with recent pneumonia literature showing that inflammation-based biomarkers may help identify patients with more severe disease or higher short-term risk, although the specific markers retained after adjustment vary by population and clinical setting (28–32). Importantly, this broader literature also supports a more nuanced interpretation rather than a single-marker conclusion: some CAP studies have highlighted the value of serial NLR (30), others have shown that multiple inflammatory indices may differ between survivors and non-survivors (31), and a recent mortality-stratification study suggested that SII may add only limited incremental value beyond established severity scores (32).

### 4.2 Interpretation of the exploratory prognostic models

The core exploratory models further supported this interpretation. When admission APACHE II score, oxygenation index, and mechanical ventilation duration were entered together with one inflammatory index, the models including NLR or SIRI provided the most favorable overall performance. These results suggest that selected inflammation-based indices may offer additional prognostic information beyond conventional severity markers, but they do not justify treating any single index as a stand-alone clinical decision tool.

The attenuation of SII and IBI after stronger adjustment is also informative. In the original analysis, multiple inflammatory indices appeared significant in relatively limited models. After more comprehensive adjustment, only SIRI and NLR retained independent associations. This indicates that confounding by illness severity and comorbidity burden was clinically meaningful in this cohort and should be considered when interpreting biomarker-outcome relationships in severe pneumonia.

### 4.3 Clinical meaning and study limitations

From a clinical perspective, SIRI and NLR may be more promising than the other evaluated indices for future external validation because they are derived from routine blood tests and remained associated with 28-day mortality after progressive adjustment. However, the present study does not establish treatment thresholds, bedside decision rules, or direct management recommendations. The findings should therefore be interpreted as exploratory and hypothesis-generating.

Several limitations should be acknowledged. This was a retrospective single-center study with 100 patients and 37 deaths, which limited the number of predictors that could be evaluated in fully adjusted models. Although we strengthened confounder control in the revised analyses, residual confounding cannot be excluded. In addition, the current results were internally explored within a single cohort and require validation in larger, multicenter populations before any broader clinical application can be considered.

## 5 Conclusion

In conclusion, among the evaluated systemic inflammatory indices, SIRI and NLR showed the most stable independent associations with 28-day all-cause mortality in severe pneumonia. These findings support the potential prognostic relevance of selected inflammation-based indices, but they should be interpreted cautiously and validated in larger prospective studies.

## Data Availability

The original contributions presented in the study are included in the article/supplementary material, further inquiries can be directed to the corresponding authors.
